# Clinical Instruction

**Published:** 1854-12

**Authors:** 


### EDITORIAL.

*Clinical Instruction.*

In a recent number of the *Journal* we made some observations on the subject of Clinical instruction, calling in question the correctness of the often repeated assertion, that we have no *true clinical teaching* in the Hospitals of this country.

It seems that our remarks called out comments in the November number of the Peninsular *Journal of Medicine*, published at Ann Arbor, Michigan. Of the nature of these comments we have no knowledge, as that number of the *Journal* has never been received by us. But in the December number of the same journal, we find an editorial explaining more fully the writer's "views upon the subject of clinical instruction, and the manner in which it should be pursued."

That we may not misrepresent, we quote from the *editorial* in question as follows, viz.:

"In order to accomplish all these purposes, we can readily see what will be necessary.

"A visit to a patient with an acute disease, once or twice a week, will by no means suffice, however careful the examination, and full the opportunity of observing, at the time, may be. Neither will a daily visit accomplish the necessary purposes, unless the student has a full opportunity for a close personal inspection of the patient, and his various conditions, and has time to enquire into the effects of the medicine—and in short, every circumstance and condition of the case. The countenance must be observed—the tongue seen—the pulse felt—the condition of the skin noticed—the chest percussed—the breathing listened to—the pupils inspected—the excretions examined, &c.

"And further than this, the management of the sick room should be studied—the conduct of the nurse, the deportment of attendants

—the whole system of management of the mind, so important a part of correct treatment—the mode of using bathing, frictions, &c., and of preparing and administering food and medicines, must receive attention

"It is also important, in true clinical instruction, that the student should see patients of a class, and under circumstances similar to those with which he will meet in practice. All must see that all these things are essential to correct clinical instruction of the highest kind.

"Now, we ask, are these circumstances possible—can these things be done in a hospital, in *any* hospital, on this or the other side of the Atlantic—in Paris, in London, in New York, in Philadelphia, in Cincinnati, or in Chicago? In the very nature of the case, the thing is impossible. In hospitals of large cities, we meet with a class of cases very different in their aspects and tendencies from those which are met with in respectable private circles, and especially from those met with in the country; and students receiving their only impressions of diseases from such sources, will be greatly at fault in their management, when they meet with cases of a class so very different, and under circumstances so completely changed."

What a pity it is that our learned editorial *confrere* at Ann Arbor, had not enlightened the profession on this subject, a few years earlier. Had it been known earlier that the "circumstances" necessary for profitable clinical instructions, were altogether "impossible" in the Hospitals, not of America alone, but in those of "London and Paris" also, it might have saved many a poor fellow the necessity of spending his last dollar to cross the ocean and gain access to the wards of those Hospitals. Again it might have prevented the assembled wisdom of our profession, as embodied in the American Medical Association, from committing so grave an error as to recommend and re-recommend from year to year, that the attendance on *Hospital* clinical instruction for at least one term, should be considered a *necessary* part of medical education, one o the requisites, indeed for admission to an examination for the degree. It is an old saying that "necessity is the mother of invention;" and who knows but the entire absence of a Hospital at Ann Arbor, constituted *the necessity* which determined the grand discovery announced in the last paragraph quoted above? But seriously, let us examine somewhat carefully the positions assumed so confidently by our worthy friend and co-laborer. And in doing so, our readers will excuse us if we leave the Hospitals of Paris, London, Philadelphia, New York, &c., to take care of themselves, while we draw our illustrations directly from the one with which we are more immediately connected at Chicago. What then are the circumstances claimed to be necessary for true and proper clinical instruction? First, the student must have opportunity for a close personal inspection of the patient. "The countenance must be observed—the tongue seen—the pulse felt—the condition of the skin noticed—the chest percussed—the breathing listened to—the pupils inspected—the excretions examined, &c." This is right, perfectly so, and just what the students visiting the wards of Mercy Hospital in Chicago, *actually do* every morning. They are brought immediately around the bed of the patient; they not only observe the countenance, but their attention is called to each lineament and item which contributes to make up the sum of its expression; they not only see the tongue, but their attention is directed to each of its peculiarities; they feel the pulse and the skin; they auscultate the chest; and they inspect the excretions. Take, for example, the actual clinical lesson for this morning. After noting briefly the progress of some cases fully examined at a previous visit, they come to the new cases for the morning. They first surround a man brought to the Hospital two days previous with typhoid fever of moderate severity. He is in the third week of the disease, having been but little interfered with by medicine of any kind. The history of his case is explicitly stated; all its diagnostic symptoms are fully examined; the changes, both general and local, which are supposed to have taken place are pointed out; then the indications for treatment, and the particular means for fulfilling them. They individually examine the countenance, skin, tongue, pulse, respiration, abdomen, and excretions. They feel of the rotund and tympanitic abdomen, and in this case see a few well marked *rose* spots.

Having finished this case they passed to another case. He too has typhoid fever, but farther advanced, tongue entirely dry, legs covered with purpuric spots, chronic abscesses forming in the cellular tissue. They examine this case with the same care as the other. Though the same form of fever, their attention is called particularly to the difference between the two, and the corresponding variations in the therapeutic indications. From this they go to a third case of the same disease. It is a young man in the early part of the second week of the disease. The fever is well marked but mild and very favorable in its progress, but they find the *rose spots* thickly covering the whole abdomen and chest. The hour is past and the class are dismissed. Here they have had the most perfect access to three cases of typhoid fever, each illustrating a particular stage and grade of the disease. They have examined them personally, their attention being specially directed to the diagnostic symptoms and pathological conditions. They will note from time to time the progress of these cases until their final termination either in convalescence or death. But before these have gone from the wards, others of the same disease will come, and during the term they can examine and compare almost every form and stage of the disease. The same thing is equally true of the other forms of fever and dysentery, as well as the various local inflammations. It is only yesterday morning that we called the attention of the last division of the class to five cases of dysentery in the same ward and almost side by side.

One was a recent and active case, two had just passed into a chronic form succeeding acute attacks, and the other two had existed three or four months presenting the extreme emaciation, enemia, and edematous legs, which accompany the long-continued ulceration of the mucous membranes. It is but a few mornings since that we had grouped together at one time and in the same ward, three cases of opthalmia, one of slight conjunctivitis another of scrofulous inflammation of the tarsus of the eye-lids, and the third severe scrofulous cornietis with ulceration. As the peculiarities of each were pointed out the diseased eyes were subjected to the direst personal examination of each student. For another clinical group we had, not long since, cases of scabies, chronic exzema of the face and erysipelas of the face all under examination and treatment at the same time. We have now in the wards patients with pulmonary tubercular deposits in almost every stage from crude primary tubercles, to complete suppuration. These have been several times carefully auscultated personally by each member of the hospital class, thereby accustoming their own ears to the variations of respitatory manner, vocal resonance, &c., which accompany this disease. The clinical lessons here alluded to are not selected ones, but simply such as have actually occurred within the last few days. They are sufficient however, to show, that the very circumstances and conditions set forth by the editor of the *Peninsular Journal* as necessary for true clinical instruction, are not only found in hospitals, but found there in a much greater degree of perfection than anywhere else in the world.

For where else is it posible to procure for the student that freedom of access to the patient for personal examination, and to such a number and variety of patients as shall illustrate not only different diseases, but also different forms and stages of the same disease? Our friend at Ann Arbor says that they are to be found "in every town, in every hamlet, in every country side' where there is an intelligent and well educated practitioner."

And again he says "*our position is, that private clinical teaching is most useful.*" Will Prof. Palmer, the author of the editorial in the Peninsular Journal, frankly tell us how many times, during his Professional life of 15 or 18 years, he has actually taken a Student to the bed-side of a respectable patient in private practice, and there detailed to him "the previous history of the disease and of the patient—the class of persons to which he belongshis individual peculiarities and tendencies—the circumstances with which the case is surrounded, &c.;" then make "a full *diagnosis* in the presence of the patient, pointing out and illustrating the means by which it is arrived at;"—also "the *special pathology* of the case—the indications of treatment, and the means by which those indications are to be fulfilled;" in the mean time allowing the student to *observe the countenance, see the tongue, feel the skin and the pulse, percuss the chest, listen to the breathing, and examine the excretions*, &c.?—And all this not once for the same patient, nor once in "*three or four days*," but *daily* from the beginning to the end of the case? Such an exercise would require the practioner to give a familiar lecture of, at least, half an hour directly over the patient. The simple statement of the question carries with it an obvious answer. Neither

Professor Palmer nor any of his colleagues have ever attempted such a task twice in their lives. They doubtless have occasionally taken a favorite pupil to see a patient with them, and allowed him to glance at the patient's tongue, feel the pulse, and look at the discharges; possibly also he has been allowed for one single time to put his ear upon the chest; and after retiring from the house a desultory conversation has been carried on in regard to the case,—we have done so, many times, and have doubtless thereby benefited to some extent our pupils. But this falls far short, in every respect, of what the Ann Arbor editor has himself described as constituting true Clinical Instruction; and equally far short of the Clinical instruction given in the Mercy Hospital every morning. It is very easy to talk about *Private* clinical instruction; and such *talk* may serve the special purpose for which it is designed, namely, to satisfy the medical students at Ann Arbor that they must look to their private preceptors, instead of the Faculty of their State University, for this most important department of medical instruction. How much of it, they will ever actually get in the several "hamlets and country rides," of Michigan, the experience of the last eighteen centuries and the common sense of every reader can easily estimate. But our friend Palmer betrays a great want of appreciation as to what *kind* of clinical instruction the student needs, when he talks about his watching "at the bed-side with *book in hand*," like a Homœopath looking for the correspondence between the symptoms of his patient and those of his supposed remedy. The very first clinical lesson he needs, is to be taught a systematic and thorough *method* of examining patients and diagnosing diseases.

He needs to be taught *how* to observe, and *what* to observe—to have pointed to him on the living patient the *import* and *value* of individual symptoms, their connections with and dependencies on each other; things which he cannot acquire except under the immediate direction of a prompt and thorough clinical teacher in the immediate presence of a variety of patients. This want of appreciation on the part of the Editor, is further shown by his quotation from Prof. Flint of Paris. To confound the *wants felt* by an experienced physician and teacher, who is abroad seeking for every thing relating to the minuter details of medical science and philosophy, with the *wants* of the student preparing to enter on the active duties of his profession, is to betray at least want of reflection. His remarks about the absence of *proper time* for attention to clinical instruction during the college terms, would have some force were it not for the fact, that so long as the present system of education continues *it must be attended to then or not at all.* But the fault here is not with the clinical instruction or the hospitals, but with the arrangement of the college terms. Few are more conscious of the defects in the present general system of medical education than we; and none have labored more diligently to remedy those defects. It will in no wise aid the good work, however, to have our Michigan friends array themselves against one of the most important departments in the whole system. It would be far better to relieve themselves at once from their false position by transferring the medical department of their University to Detroit, where they could do justice to the students of their own state by having access to Hospitals. If this was done we should hear no more about the worthlessness of Hospital Clinical Instruction.

D.

---

The caption to the first article in the present number of the journal, should have stated that the paper of Dr. Thompson was read before the Esculapian Medical Society, and forwarded for publication by the Secretary in accordance with a vote of the Society.

The article we promised to give in this number, on Epidemic Jaundice, is deferred to make room for other communications.

---

*Close of the Volume :*—The present number closes the third volume, of the New-Series of the North-Western Medical and Surgical Journal. The Journal has for several years been established on a firm basis, and has a wide circulation.

Owing to some temporary obstacles, the issue of the Journal has been more irregular during the past year, than we could have wished.

This source of annoyance however, will cease with the commencement of the new Volume. The present is a favorable time for subscribers who are in arrears, to pay up, and for new ones to forward their names and their money.

But hereafter all communications and letters relating to the Journal should be directed to Dr. N. S. Davis, Chicago, Ill.

